# Dimethyl 1-(2-cyano­benz­yl)-1*H*-pyrazole-3,5-dicarboxyl­ate

**DOI:** 10.1107/S1600536809015153

**Published:** 2009-04-30

**Authors:** Ji-Yuan Yao, Jie Xiao, Hong Zhao

**Affiliations:** a Ordered Matter Science Research Center, College of Chemistry and Chemical Engineering, Southeast University, Nanjing 210096, People’s Republic of China

## Abstract

In the mol­ecule of the title compound, C_15_H_13_N_3_O_4_, the dihedral angle between the pyrazole and benzene rings is 79.89 (6)°. An intra­molecular C—H⋯O hydrogen bond is present. The crystal structure is stabilized by π–π stacking inter­actions between centrosymmetrically related pyrazole rings with a centroid–centroid distance of 3.500 (3) Å.

## Related literature

For the use of pyrazoles as ligands, see: Dvorak *et al.* (2005 ▶). For the use of nitrile derivatives in the synthesis of heterocyclic compounds, see: Radl *et al.* (2000 ▶). For a related structure, see: Fu & Zhao (2007 ▶).
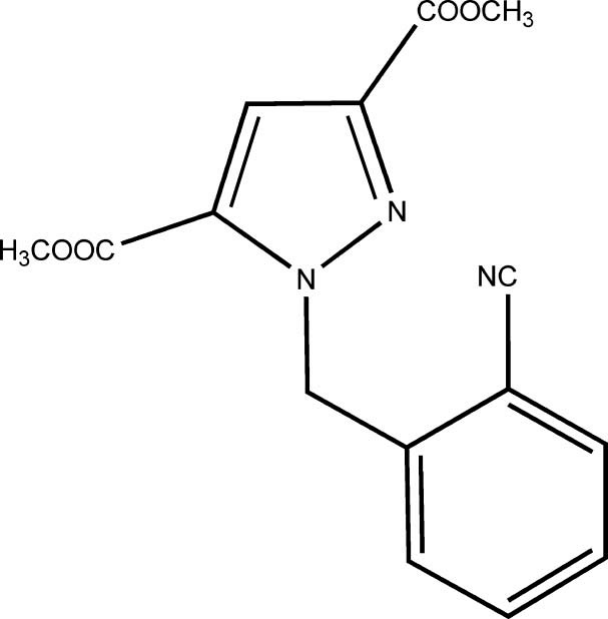


## Experimental

### 

#### Crystal data

C_15_H_13_N_3_O_4_
*M* *_r_* = 299.28
Monoclinic, 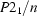

*a* = 7.2416 (19) Å
*b* = 10.977 (3) Å
*c* = 18.405 (4) Å
β = 100.670 (11)°
*V* = 1437.7 (6) Å^3^
*Z* = 4
Mo *K*α radiation
μ = 0.10 mm^−1^
*T* = 291 K
0.35 × 0.30 × 0.25 mm

#### Data collection

Rigaku SCXmini diffractometer
Absorption correction: multi-scan (*CrystalClear*; Rigaku, 2005 ▶) *T*_min_ = 0.968, *T*_max_ = 0.980
14431 measured reflections
3287 independent reflections
2452 reflections with *I* > 2σ(*I*)
*R*_int_ = 0.040

#### Refinement

*R*[*F* ^2^ > 2σ(*F* ^2^)] = 0.052
*wR*(*F* ^2^) = 0.130
*S* = 1.09
3287 reflections
201 parameters
H-atom parameters constrained
Δρ_max_ = 0.18 e Å^−3^
Δρ_min_ = −0.19 e Å^−3^

### 

Data collection: *CrystalClear* (Rigaku, 2005 ▶); cell refinement: *CrystalClear*; data reduction: *CrystalClear*; program(s) used to solve structure: *SHELXS97* (Sheldrick, 2008 ▶); program(s) used to refine structure: *SHELXL97* (Sheldrick, 2008 ▶); molecular graphics: *SHELXTL/PC* (Sheldrick, 2008 ▶); software used to prepare material for publication: *SHELXTL/PC*.

## Supplementary Material

Crystal structure: contains datablocks I, global. DOI: 10.1107/S1600536809015153/rz2312sup1.cif

Structure factors: contains datablocks I, I. DOI: 10.1107/S1600536809015153/rz2312Isup2.hkl

Additional supplementary materials:  crystallographic information; 3D view; checkCIF report

## Figures and Tables

**Table 1:** Hydrogen-bond geometry (Å, °)

*D*—H⋯*A*	*D*—H	H⋯*A*	*D*⋯*A*	*D*—H⋯*A*
C8—H8*A*⋯O3	0.97	2.41	2.917 (2)	112

**Fig. 1.**
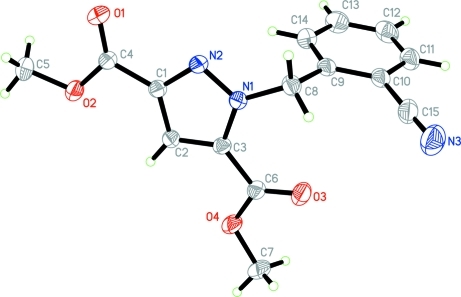
The molecular structure of the title compound, showing the atomic numbering scheme. Displacement ellipsoids are drawn at the 30% probability level.

## supplementary crystallographic information

### Comment

Pyrazoles are considered as extremely versatile building blocks in organic chemistry. They constitute key fragments in active pharmaceutical and agrochemical ingredients, which have found widespread use as ligands for transition metals (Dvorak *et al.*, 2005), In addition, nitrile derivatives are important materials in the synthesis of some heterocyclic molecules (Radl *et al.*, 2000). Recently, we have reported a few benzonitrile compounds (Fu & Zhao, 2007). As an extension of our work on the structural characterization of nitrile compounds, the structure of the title compound is reported here.

In the molecule of the title compound (Fig. 1) bond lengths and angles have normal values. The dihedral angle between the planes of the pyrazole and phenyl rings is 79.89 (6) °. The molecular conformation is stabilized by an intramolecular C—H···O hydrogen bond (Table 1). In the crystal packing, centrosymmetrically related molecules at (x, y, z) and (2-x, -y, -z) are connected by a π-π stacking interaction involving the pyrazole rings, with a centroid-centroid separation of 3.500 (3) Å, a perpendicular interplanar distance of 3.382 (3) and a centroid-centroid offset of 0.901 (2) Å.

### Experimental

1*H*-Pyrazole-3,5-dicarboxylic acid dimethyl ester (0.185 mg, 1 mmol) and 2-(bromomethyl)benzonitrile (0.196 mg, 1 mmol) were dissolved in acetone in the presence of K_2_CO_3_ (0.138 mg, 1 mmol) and heated under reflux for 1 day. After the mixture was cooled to room temperature, the solution was filtered and the solvents removed in vacuum to afford a white precipitate of the title compound. Colourless crystals suitable for X-ray diffraction were obtained after 9 days by slow evaporation of a diethylether solution.

### Refinement

All H atoms were detected in a difference Fourier map, but were placed in calculated positions and refined using a riding motion approximation, with C—H = 0.93–0.97 Å and with *U*_iso_(H) = 1.2 *U*_eq_(C) or 1.5 *U*_eq_(C) for methyl H atoms.

### Crystal data

**Table:**

C_15_H_13_N_3_O_4_	*F*(000) = 624
*M**_r_* = 299.28	*D*_x_ = 1.383 Mg m^−^^3^
Monoclinic, *P*2_1_/*n*	Mo *K*α radiation, λ = 0.71073 Å
Hall symbol: -P 2yn	Cell parameters from 3210 reflections
*a* = 7.2416 (19) Å	θ = 2.9–27.5°
*b* = 10.977 (3) Å	µ = 0.10 mm^−^^1^
*c* = 18.405 (4) Å	*T* = 291 K
β = 100.670 (11)°	Prism, colourless
*V* = 1437.7 (6) Å^3^	0.35 × 0.30 × 0.25 mm
*Z* = 4	

### Data collection

**Table:**

Rigaku SCXmini diffractometer	3287 independent reflections
Radiation source: fine-focus sealed tube	2452 reflections with *I* > 2σ(*I*)
graphite	*R*_int_ = 0.040
Detector resolution: 13.6612 pixels mm^-1^	θ_max_ = 27.5°, θ_min_ = 2.9°
ω scans	*h* = −9→9
Absorption correction: multi-scan (*CrystalClear*; Rigaku, 2005)	*k* = −14→14
*T*_min_ = 0.968, *T*_max_ = 0.980	*l* = −23→23
14431 measured reflections	

### Refinement

**Table:**

Refinement on *F*^2^	Primary atom site location: structure-invariant direct methods
Least-squares matrix: full	Secondary atom site location: difference Fourier map
*R*[*F*^2^ > 2σ(*F*^2^)] = 0.052	Hydrogen site location: inferred from neighbouring sites
*wR*(*F*^2^) = 0.130	H-atom parameters constrained
*S* = 1.09	*w* = 1/[σ^2^(*F*_o_^2^) + (0.0618*P*)^2^ + 0.1406*P*] where *P* = (*F*_o_^2^ + 2*F*_c_^2^)/3
3287 reflections	(Δ/σ)_max_ < 0.001
201 parameters	Δρ_max_ = 0.18 e Å^−^^3^
0 restraints	Δρ_min_ = −0.19 e Å^−^^3^

### Special details

**Table:**

Geometry. All e.s.d.'s (except the e.s.d. in the dihedral angle between two l.s. planes) are estimated using the full covariance matrix. The cell e.s.d.'s are taken into account individually in the estimation of e.s.d.'s in distances, angles and torsion angles; correlations between e.s.d.'s in cell parameters are only used when they are defined by crystal symmetry. An approximate (isotropic) treatment of cell e.s.d.'s is used for estimating e.s.d.'s involving l.s. planes.
Refinement. Refinement of *F*^2^ against ALL reflections. The weighted *R*-factor *wR* and goodness of fit *S* are based on *F*^2^, conventional *R*-factors *R* are based on *F*, with *F* set to zero for negative *F*^2^. The threshold expression of *F*^2^ > σ(*F*^2^) is used only for calculating *R*-factors(gt) *etc*. and is not relevant to the choice of reflections for refinement. *R*-factors based on *F*^2^ are statistically about twice as large as those based on *F*, and *R*- factors based on ALL data will be even larger.

### Fractional atomic coordinates and isotropic or equivalent isotropic displacement parameters (Å^2^)

**Table:**

	*x*	*y*	*z*	*U*_iso_*/*U*_eq_	
C1	0.7452 (2)	−0.11741 (14)	−0.00030 (8)	0.0384 (4)	
C2	0.7207 (2)	0.00398 (14)	−0.02209 (9)	0.0384 (4)	
H2	0.6593	0.0337	−0.0675	0.046*	
C3	0.8066 (2)	0.07034 (14)	0.03796 (9)	0.0369 (4)	
C4	0.6825 (2)	−0.22943 (15)	−0.04182 (9)	0.0430 (4)	
C5	0.5284 (3)	−0.3033 (2)	−0.15741 (12)	0.0698 (6)	
H5A	0.6333	−0.3544	−0.1618	0.105*	
H5B	0.4715	−0.2736	−0.2054	0.105*	
H5C	0.4378	−0.3494	−0.1368	0.105*	
C6	0.8293 (2)	0.20236 (15)	0.04805 (9)	0.0419 (4)	
C7	0.7579 (3)	0.39182 (17)	−0.01060 (12)	0.0691 (6)	
H7A	0.6940	0.4259	0.0259	0.104*	
H7B	0.7038	0.4234	−0.0584	0.104*	
H7C	0.8886	0.4132	0.0009	0.104*	
C8	0.9833 (2)	0.00848 (16)	0.16631 (9)	0.0438 (4)	
H8A	1.0696	0.0758	0.1655	0.053*	
H8B	1.0572	−0.0635	0.1826	0.053*	
C9	0.8571 (2)	0.03652 (14)	0.22102 (9)	0.0409 (4)	
C10	0.9142 (2)	0.11795 (15)	0.27911 (9)	0.0455 (4)	
C11	0.8042 (3)	0.13819 (17)	0.33260 (10)	0.0572 (5)	
H11	0.8447	0.1919	0.3714	0.069*	
C12	0.6365 (3)	0.0788 (2)	0.32785 (11)	0.0639 (5)	
H12	0.5623	0.0924	0.3633	0.077*	
C13	0.5775 (3)	−0.0008 (2)	0.27081 (12)	0.0636 (5)	
H13	0.4631	−0.0408	0.2677	0.076*	
C14	0.6869 (3)	−0.02201 (17)	0.21785 (10)	0.0519 (4)	
H14	0.6452	−0.0764	0.1796	0.062*	
C15	1.0869 (3)	0.18543 (18)	0.28545 (10)	0.0560 (5)	
N1	0.87843 (18)	−0.01204 (12)	0.09157 (7)	0.0385 (3)	
N2	0.84141 (19)	−0.12686 (12)	0.06921 (7)	0.0414 (3)	
N3	1.2207 (3)	0.24250 (19)	0.29236 (11)	0.0811 (6)	
O1	0.7088 (2)	−0.33067 (12)	−0.01849 (7)	0.0649 (4)	
O2	0.5924 (2)	−0.20179 (11)	−0.10967 (7)	0.0574 (4)	
O3	0.9169 (2)	0.25110 (12)	0.10214 (7)	0.0645 (4)	
O4	0.7390 (2)	0.26063 (10)	−0.01091 (7)	0.0568 (4)	

### Atomic displacement parameters (Å^2^)

**Table:**

	*U*^11^	*U*^22^	*U*^33^	*U*^12^	*U*^13^	*U*^23^
C1	0.0436 (9)	0.0354 (8)	0.0381 (9)	0.0005 (7)	0.0124 (7)	0.0002 (6)
C2	0.0426 (9)	0.0358 (8)	0.0364 (8)	0.0020 (6)	0.0068 (7)	−0.0002 (6)
C3	0.0405 (8)	0.0315 (8)	0.0404 (8)	0.0021 (6)	0.0121 (7)	0.0012 (6)
C4	0.0520 (10)	0.0354 (9)	0.0437 (9)	−0.0026 (7)	0.0140 (8)	−0.0022 (7)
C5	0.0987 (17)	0.0559 (12)	0.0525 (12)	−0.0175 (11)	0.0084 (11)	−0.0181 (9)
C6	0.0468 (9)	0.0367 (9)	0.0433 (9)	0.0000 (7)	0.0112 (7)	−0.0030 (7)
C7	0.1012 (17)	0.0315 (10)	0.0721 (14)	−0.0033 (10)	0.0092 (12)	0.0056 (9)
C8	0.0463 (9)	0.0459 (10)	0.0377 (9)	0.0035 (7)	0.0041 (7)	−0.0006 (7)
C9	0.0487 (9)	0.0368 (8)	0.0366 (8)	0.0070 (7)	0.0058 (7)	0.0040 (6)
C10	0.0579 (10)	0.0394 (9)	0.0377 (9)	0.0082 (7)	0.0050 (8)	0.0025 (7)
C11	0.0759 (13)	0.0531 (12)	0.0438 (10)	0.0114 (9)	0.0148 (10)	−0.0049 (8)
C12	0.0738 (14)	0.0707 (14)	0.0537 (11)	0.0150 (11)	0.0288 (10)	0.0053 (10)
C13	0.0589 (12)	0.0713 (14)	0.0646 (13)	−0.0013 (10)	0.0220 (10)	0.0057 (10)
C14	0.0577 (11)	0.0501 (11)	0.0484 (10)	−0.0008 (8)	0.0114 (9)	−0.0012 (8)
C15	0.0632 (12)	0.0537 (12)	0.0484 (10)	0.0007 (9)	0.0030 (9)	−0.0093 (8)
N1	0.0453 (8)	0.0350 (7)	0.0356 (7)	0.0015 (5)	0.0089 (6)	−0.0003 (5)
N2	0.0523 (8)	0.0324 (7)	0.0407 (7)	0.0006 (6)	0.0122 (6)	0.0003 (5)
N3	0.0735 (13)	0.0847 (14)	0.0806 (14)	−0.0193 (11)	0.0024 (10)	−0.0156 (11)
O1	0.0995 (11)	0.0333 (7)	0.0601 (8)	−0.0036 (7)	0.0103 (8)	0.0019 (6)
O2	0.0811 (9)	0.0403 (7)	0.0458 (7)	−0.0055 (6)	−0.0011 (6)	−0.0065 (5)
O3	0.0864 (10)	0.0431 (7)	0.0573 (8)	−0.0058 (7)	−0.0037 (7)	−0.0088 (6)
O4	0.0792 (9)	0.0307 (6)	0.0555 (8)	0.0007 (6)	−0.0005 (7)	0.0026 (5)

### Geometric parameters (Å, °)

**Table:**

C1—N2	1.343 (2)	C7—H7C	0.9600
C1—C2	1.393 (2)	C8—N1	1.460 (2)
C1—C4	1.474 (2)	C8—C9	1.512 (2)
C2—C3	1.373 (2)	C8—H8A	0.9700
C2—H2	0.9300	C8—H8B	0.9700
C3—N1	1.368 (2)	C9—C14	1.381 (3)
C3—C6	1.466 (2)	C9—C10	1.396 (2)
C4—O1	1.194 (2)	C10—C11	1.394 (3)
C4—O2	1.332 (2)	C10—C15	1.440 (3)
C5—O2	1.441 (2)	C11—C12	1.367 (3)
C5—H5A	0.9600	C11—H11	0.9300
C5—H5B	0.9600	C12—C13	1.372 (3)
C5—H5C	0.9600	C12—H12	0.9300
C6—O3	1.202 (2)	C13—C14	1.385 (3)
C6—O4	1.324 (2)	C13—H13	0.9300
C7—O4	1.446 (2)	C14—H14	0.9300
C7—H7A	0.9600	C15—N3	1.141 (3)
C7—H7B	0.9600	N1—N2	1.3376 (18)
			
N2—C1—C2	111.35 (14)	C9—C8—H8A	109.1
N2—C1—C4	119.02 (14)	N1—C8—H8B	109.1
C2—C1—C4	129.63 (15)	C9—C8—H8B	109.1
C3—C2—C1	105.13 (14)	H8A—C8—H8B	107.8
C3—C2—H2	127.4	C14—C9—C10	117.76 (16)
C1—C2—H2	127.4	C14—C9—C8	121.46 (15)
N1—C3—C2	106.57 (13)	C10—C9—C8	120.69 (16)
N1—C3—C6	122.89 (14)	C11—C10—C9	121.04 (18)
C2—C3—C6	130.53 (15)	C11—C10—C15	117.53 (17)
O1—C4—O2	124.53 (16)	C9—C10—C15	121.42 (16)
O1—C4—C1	125.21 (16)	C12—C11—C10	119.74 (18)
O2—C4—C1	110.25 (14)	C12—C11—H11	120.1
O2—C5—H5A	109.5	C10—C11—H11	120.1
O2—C5—H5B	109.5	C11—C12—C13	119.98 (18)
H5A—C5—H5B	109.5	C11—C12—H12	120.0
O2—C5—H5C	109.5	C13—C12—H12	120.0
H5A—C5—H5C	109.5	C12—C13—C14	120.5 (2)
H5B—C5—H5C	109.5	C12—C13—H13	119.7
O3—C6—O4	124.65 (16)	C14—C13—H13	119.7
O3—C6—C3	125.05 (16)	C9—C14—C13	120.93 (18)
O4—C6—C3	110.30 (14)	C9—C14—H14	119.5
O4—C7—H7A	109.5	C13—C14—H14	119.5
O4—C7—H7B	109.5	N3—C15—C10	177.0 (2)
H7A—C7—H7B	109.5	N2—N1—C3	111.90 (13)
O4—C7—H7C	109.5	N2—N1—C8	118.35 (13)
H7A—C7—H7C	109.5	C3—N1—C8	129.75 (14)
H7B—C7—H7C	109.5	N1—N2—C1	105.04 (12)
N1—C8—C9	112.67 (14)	C4—O2—C5	116.21 (14)
N1—C8—H8A	109.1	C6—O4—C7	116.47 (14)

### Hydrogen-bond geometry (Å, °)

**Table:**

*D*—H···*A*	*D*—H	H···*A*	*D*···*A*	*D*—H···*A*
C8—H8A···O3	0.97	2.41	2.917 (2)	112
